# Investigations on the Respiratory Function in COVID-19 Patients: A Prospective Cohort Study

**DOI:** 10.1155/2021/9928276

**Published:** 2021-12-26

**Authors:** Moyi Li, Congyang Zhou, Jian Jiang, Huangjun You, Chongchong Liu, Peng Shen, Zhen Feng

**Affiliations:** ^1^Department of Rehabilitation Medicine, The First Affiliated Hospital of Nanchang University, Nanchang 330006, China; ^2^Department of Emergency Medicine, The First Affiliated Hospital of Nanchang University, Nanchang 330006, China

## Abstract

**Introduction:**

Coronavirus disease 2019 (COVID-19) is a global public health crisis. However, whether it can cause respiratory dysfunction or physical and psychological disorders in patients remains unknown. Thus, this study was conducted to investigate the respiratory function, activities of daily living, quality of life, and mental status of patients with COVID-19. *Participants and outcomes*. Data was collected from the follow-up of eligible patients who attended the fever clinic of three hospitals in Jiangxi Province, from March to May 2020. The outcomes included respiratory muscle function, degree of dyspnea, aerobic capacity, activities of daily living, quality of life, and mental status.

**Results:**

A total of 139 patients (72 men and 67 women) were included in this study. The proportions of mild, moderate, severe, and critical cases of COVID-19 were 7.1% (10 cases), 68.3% (95 cases), 20.1% (28 cases), and 4.2% (6 cases), respectively. The rates of abnormal maximal inspiratory pressure were 10.0%, 25.2%, 25.0%, and 16.7%, respectively. There were 50%, 65.3%, 50%, and 66.7% of the patients with abnormal dyspnea in the four clinical classifications, respectively. Patients generally show a decline in quality of life, anxiety, and depression symptoms.

**Conclusions:**

Respiratory dysfunction, decreased quality of life, and psychological disorders were present in each clinical classification of COVID-19. Therefore, it is necessary to carry out respiratory rehabilitation and psychological intervention for COVID-19 patients.

## 1. Introduction

Since December 2019, coronavirus disease 2019 (COVID-19) has become a public health emergency and has spread to almost all countries worldwide. The current epidemic situation is still very serious. On March 11, the World Health Organization (WHO) held a press conference in Geneva and officially announced that COVID-19 was a global pandemic [1]. Since then, the number of confirmed cases and deaths worldwide has been rising. The latest situation report released by the WHO showed that the total number of confirmed cases and deaths was more than 117 million and 2.6 million, respectively, as of March 11, 2021 [2].

COVID-19 has already been classified as a category B infectious disease according to the Law of the People's Republic of China on the Prevention and Treatment of Infectious Diseases, and control measures for category A infectious diseases have been adopted. The National Health Commission has published diagnosis and treatment protocols to guide clinical diagnosis and treatment. With the accumulating experience of treating COVID-19 patients, particularly those classified as severe and critical in clinical practice, our understanding of COVID-19 has been continuously deepened. Standardized respiratory rehabilitation techniques and procedures in various regions are essential for patients with varying degrees of respiratory, physical, and psychological dysfunction [3].

With the continuous deepening of the understanding of this disease, the “Notice on Printing and Distributing the Diagnosis and Treatment Program of COVID-19” was promulgated by the General Office of the National Health Commission and the Office of the State Administration of Traditional Chinese Medicine and has been updated in the seventh edition. The diagnosis and treatment of COVID-19 has achieved encouraging results in China and other countries with COVID-19 response similar to that of China and benefit from that. Updated data showed that the number of cured cases globally was nearly 92 million [3]. However, discharged patients may experience varying degrees of disorders of respiratory function, activities of daily living (ADL), and psychological function [4]. Evidence of reduced ADL and negative emotions in adults with impaired respiratory function has been given, which can affect their quality of life (QOL) and restrict their return to family and society [5].

As a subtype of coronavirus, the COVID-19 virus has the closest genetic relationship with the severe acute respiratory syndrome (SARS) coronavirus, sharing 79.6% sequence identity and similar clinical symptoms [6]. Previous studies reported that SARS survivors had deficits in cardiorespiratory and musculoskeletal performance, and their health-related QOL appeared to be significantly impaired [7].

Considering COVID-19 is a new disease, there are no reports on its effects on the respiratory function, physical fitness, and QOL of recovering patients. This is important because knowing the effects of COVID-19 on these parameters may guide the design of rehabilitation programs and assessments. Therefore, this study is aimed at evaluating the respiratory and QOL-related functions of patients recovering from COVID-19 in the subacute and stable phases.

## 2. Materials and Methods

### 2.1. Participants

From March to May 2020, follow-ups were conducted at the COVID-19 fever clinics of the First Affiliated Hospital of Nanchang University, the First People's Hospital of Jiujiang City, and the People's Hospital of Xinyu City, Jiangxi Province. A total of 139 patients met the inclusion criteria and were included during the study period; the respiratory function, QOL, and mental status of each subject were evaluated after the subjects were discharged from the hospital.

### 2.2. Inclusion Criteria

Patients

1. Who were diagnosed with COVID-19 (in accordance with the diagnostic criteria of the Diagnosis and Treatment Program of COVID-19 (7th Version of Trial Implementation) [3] issued by the National Health Commission of the People's Republic of China) and met the discharge criteria
2. Older than 18 years old
3. Who were conscious
4. Volunteered to participate in the study and sign the informed consent form

### 2.3. Exclusion Criteria

Patients with

1. Unstable vital signs
2. Severe primary disease of the liver, kidney, hematological, and endocrine system
3. History of psychosis, substance abuse, or dependence

Inclusion and exclusion criteria evaluations were performed by three independent and trained doctors who worked in the fever clinics of the three hospitals.

### 2.4. Assessment

1. Respiratory muscle function: maximal inspiratory pressure (MIP), which is the most commonly used and most useful measure to evaluate inspiratory muscle function [8], was measured using the POWERbreathe inspiratory muscle assessment system (produced by POWERbreathe International Ltd., England). Due to the high transmissibility of the COVID-19, we performed this assessment in the isolation clinic rooms
2. Degree of dyspnea: the modified Medical Research Council dyspnea index scale (mMRC) was assessed using the corresponding scores. mMRC is a simple, useful, and practical 5-point scale to assess the degree of dyspnea [9]
3. Aerobic capacity: the 6-min walk test (6MWT) was used as the outcome of the patients' aerobic capacity. The 6MWT is the most useful tool to reflect the level of functional compensatory ability to complete daily physical activities [10]. The test was performed in a wide flat space in the hospitals under adequate protection
4. Activities of daily living: the Barthel Index (BI) was used for the evaluation. The BI is a widely used scale for the functional assessment of independence level in various diseases, and its validity and reliability have been verified [11]
5. QOL: The MOS item short from the health survey (SF-36) was used to evaluate the patients' QOL. The SF-36 consists of 36 questions, with a total score ranging from 0 to 100, which is computed for eight areas and the physical and mental summary scores. Higher value indicates a better health status [12]
6. Mental status: Hamilton Anxiety Scale (HAMA) and Hamilton Depression Scale (HAMD) were used to evaluate the patients' anxiety and depression status, respectively. HAMA and HAMD have been verified high reliability and validity to assess the severity of depression and anxiety symptoms [13]

### 2.5. Statistical Analysis

Analyses were performed using SPSS 21.0 (IBM, Chicago, IL, USA) software packages. Statistical significance was defined as a two-sided *P* value of <0.05.

In the descriptive analysis of the sample, continuous variables are expressed using mean and standard deviation for normal distribution and median and interquartile range for non-normal distribution. The Kolmogorov-Smirnov test was used to test for normality. Appropriate transformations were applied in the case of a nonnormal distribution. Categorical variables are expressed as proportions with their standard error. For comparison of the assessment measurements between mild, moderate, severe, and critical cases, a *t*-test or nonparametric tests was used for continuous data and Pearson chi-squared or Fisher exact test for categorical data.

## 3. Results

A total of 318 confirmed COVID-19 patients were admitted to the First Affiliated Hospital of Nanchang University, the First People's Hospital of Jiujiang City, and the People's Hospital of Xinyu City, Jiangxi Province, but 179 patients were not involved in the study. Among them, 101 patients returned to their hometowns for quarantine after being discharged from the hospital and were unable to participate in the study, and 24 patients lost touch and 54 patients refused to participate in the study. As a result, the remaining 139 patients (72 males and 67 females) were included in the study. The average age of the patients was 44.0 years (range, 18–85; standard deviation, 13.5).

The proportions of mild, moderate, severe, and critical cases of COVID-19 were 7.1% (10 cases), 68.3% (95 cases), 20.1% (28 cases), and 4.2% (6 cases), respectively. The proportions of abnormal MIP (50% lower than expected) [14] were 10.0%, 25.2%, 25.0%, and 16.7% in the mild, moderate, severe, and critical cases, respectively. In the four clinical categories, there were 50%, 65.3%, 50%, and 66.7% of patients with abnormal dyspnea (mMRC score > 0). Only two severe and two critical patients were rated as grade 4 (most serious). As the clinical severity of the disease increases, patients' ADL shows a decreasing trend. The baseline characteristics and respiratory functions of all the subjects are listed in Table 1.

The scores for SF-36 for each domain are shown in Table 2. In mild, moderate, severe, and critical cases, physical functioning and role-physical scores showed a gradual decline. On the contrary, the scores for general health and social function increased in the four classifications.

The results showed that at least 17% of each classification was in anxious or depressive status, and the highest proportion of abnormal anxiety and depression status was found in critically ill patients (33.3% both). The details are presented in Table 3.

## 4. Discussion

In this study, confirmed cases (*n* = 139) with available data and included in the analysis were classified as mild (*n* = 10, 7.1%), moderate (*n* = 95, 68.3%), severe (*n* = 28, 20.1%), and critical (*n* = 6, 4.2%), and the ratios were similar to those reported recently [15]. Among the 139 cases, the number of male patients (72, 51.8%) was almost equal to that of female patients (67, 48.2%). This is consistent with the situation in mild/moderate cases. However, there were significantly more male than female patients in severe/critical cases, which is consistent with previous reports [16, 17].

The MIP results showed that the respiratory muscle strength of severe and critical cases was greater than that of mild and moderate cases. This might be due to the significantly higher proportion of male patients in severe and critical cases than mild and moderate patients. Furthermore, in mild (10%), moderate (25.2%), and severe (25%) cases, the proportion of abnormal MIP increased roughly with the severity of the disease, but only 16.7% of critically ill patients with abnormal MIP. The reason might be that the six critical cases took a long time from discharge to investigation, and their respiratory muscle strength improved during this rehabilitation period.

Each clinical classification had an abnormal degree of dyspnea, but only two severe and two critical patients were assessed as the most serious. Pathological results from a study in which biopsy samples obtained from a COVID-19 death case showed bilateral diffuse alveolar damage with cellular fibromyxoid exudates, interstitial mononuclear inflammatory infiltrates, desquamation of pneumocytes, pulmonary edema, and hyaline membrane formation in lung tissue. This indicated that the patient had acute respiratory distress syndrome, which led to dyspnea and respiratory dysfunction [18]. In the Diagnosis and Treatment Program of COVID-19 (7th Version of Trial Implementation), it indicated that the coronavirus can cause not only pathological changes in the lungs but also damage to multiple organs, including the spleen, heart and blood vessels, liver and gallbladder, and kidneys, which decrease aerobic capacity and physical function and even reduce ADL and QOL, as well as lead to dysfunction in mental health [3].

The results of the current investigation suggest that even mild and moderate cases have respiratory dysfunction, which reveals that respiratory rehabilitation is necessary in both severe/critical and mild/moderate cases. Up to now, there is few clinical research report the respiratory function status of COVID-19 after discharge. Previous studies have recently shown that respiratory training can improve the respiratory function of COVID-19 patients and reduce the symptoms of dyspnea and respiratory weakness [19, 20]. A review reports that physical and psychological changes might exist in COVID-19 patients after discharge, such as reduction in respiratory muscle strength, diffusing lung capacity for carbon monoxide, endurance and quality of life, and increase in anxiety and depression symptoms, while respiratory muscle training, cough exercise, stretching exercise, and home exercise have resulted in improvement in those functions and symptoms [21]. However, these studies did not define whether there were disorders in the patients' respiratory function, ADL, quality of life, and mental status.

Holmes et al. [22] explored the psychological, social, and neuroscientific effects of COVID-19 in the UK and found that anxiety, depression, stress, and other negative emotions increased, especially in infected people. Therefore, they called for action to provide urgent and long-term strategies for mental health science research. We also found that many patients had potentially anxious or depressive status. This suggests that it is important to intervene psychologically.

The findings of this study can help us understand the respiratory function, psychological condition, and QOL of COVID-19 patients and may be valuable in the current efforts to fight the global pandemic.

## 5. Limitations

This study has several limitations. First, due to the small sample size, the number of patients limited the accuracy of the results, especially for mild and critical classifications. Future research could increase the sample size to verify our results. Second, due to the particularity of the disease, plenty of medical examinations (e.g., pulmonary function tests) have not been implemented, resulting in the limitation of the comprehensiveness of the results. Third, more examinations related to respiratory function should be performed in COVID-19 patients under the premise of safety in the future.

## 6. Conclusions

Respiratory dysfunction, decreased QOL, and psychological disorders exist in each clinical classification of COVID-19. Therefore, it is necessary to carry out respiratory rehabilitation and psychological intervention for COVID-19 patients.

## Figures and Tables

**Table 1:** Baseline characteristics and respiratory function of the 139 patients.

Characteristics	Mild (*N* = 10)	Moderate (*N* = 95)	Severe (*N* = 28)	Critical (*N* = 6)	Total (*N* = 139)
Age, mean (SD) (y)	48.6 (18.7)	41.9 (11.7)	48.0 (13.7)	50.7 (22.3)	44.0 (13.5)
Sex					
Male (*N*, %)	6, 60.0%	42, 44.2%	19, 67.9%	5, 83.33%	72, 51.8%
Female (*N*, %)	4, 40.0%	53, 55.8%	9, 32.1%	1, 16.67%	67, 48.2%
Height, mean (SD) (cm)	165.4 (6.7)	163.7 (6.8)	168.1 (5.56)	172.8 (3.77)	165.1 (6.8)
Weight, mean (SD) (kg)	68.6 (9.5)	63.4 (11.7)	69.1 (10.3)	75.0 (14.2)	65.5 (11.6)
MIP, mean (SD) (cmH_2_O)	75.9 (25.3)	69.7 (30.7)	75.5 (36.0)	91.9 (41.0)	72.3 (32.0)
mMRC					
0 (*N*, %)	5, 50.0%	62, 65.3%	14, 50.0%	4, 66.7%	85, 61.2%
1 (*N*, %)	5, 50.0%	27, 28.4%	11, 39.3%	0, 0	43, 30.9%
2 (*N*, %)	0, 0	5, 5.3%	1, 3.6%	0, 0	6, 4.3%
3 (*N*, %)	0, 0	1, 1.0%	0, 0	0, 0	1,0.7%
4 (*N*, %)	0, 0	0, 0	2, 7.1%	2, 33.3%	4, 2.9%
6-min walk distance, mean (SD) (m)	509.5 (115.8)	547.5 (86.8)	550.3 (84.0)	625.9 (63.2)	547.5 (88.8)
BI, mean (SD)	100.0 (0)	100.0 (0)	98.4 (8.5)	81.7 (29.1)	98.9 (7.7)

**Table 2:** QOL outcomes of the 139 patients [mean (SD)].

Domains	Mild (*N* = 10)	Moderate (*N* = 95)	Severe (*N* = 28)	Critical (*N* = 6)	Total (*N* = 139)
PF	86.0 (10.2)	85.3 (13.2)	83.0 (23.2)	60.0 (46.6)	83.8 (18.4)
RF	62.5 (48.9)	54.4 (40.7)	35.7 (34.3)	37.5 (49.4)	50.4 (40.8)
BP	73.6 (16.2)	80.8 (15.1)	79.9 (17.9)	85.3 (7.7)	80.3 (15.5)
GH	45.6 (8.6)	48.6 (11.3)	50.3 (7.7)	57.3 (16.6)	49.1 (10.9)
VT	67.0 (17.7)	76.2 (15.5)	78.0 (13.4)	78.3 (15.1)	76.0 (15.3)
SF	36.7 (35.8)	53.8 (28.8)	67.9 (31.0)	70.4 (35.6)	56.1 (30.8)
RE	76.7 (21.0)	80.5 (38.0)	85.7 (29.3)	66.7 (51.6)	80.7 (37.1)
MH	76.4 (21.0)	77.1 (16.5)	85.4 (10.1)	76.7 (25.2)	78.7 (16.4)
HT	25.0 (20.4)	34.0 (20.7)	34.8 (17.1)	29.2 (29.2)	33.3 (20.3)

PF: physical functioning; RP: role-physical; BP: bodily pain; GH: general health; VT: vitality; SF: social functioning; RE: role-emotional; MH: mental health; HT: reported health transition.

**Table 3:** Mental status of the 139 patients.

	Mild (*N* = 10)	Moderate (*N* = 95)	Severe (*N* = 28)	Critical (*N* = 6)	Total (*N* = 139)
HAMA					
Mean (SD)	6.4 (7.4)	5.7 (5.8)	5.1 (6.1)	5.7 (8.0)	5.6 (6.0)
Abnormal, *N* (%)	3, 30.0%	27, 28.4%	5, 17.9%	2, 33.3%	37, 26.6%
HAMD					
Mean (SD)	4.8 (4.4)	6.1 (5.0)	5.0 (4.6)	7.2 (8.6)	5.8 (5.0)
Abnormal, *N* (%)	2, 20.0%	28, 29.5%	7, 25%	2, 33.3%	39, 28.1%

## Data Availability

All data generated or used during the study appear in the submitted article.
